# SARS-CoV-2 Genomic Surveillance from Community-Distributed Rapid Antigen Tests, Wisconsin, USA

**DOI:** 10.3201/eid3113.241192

**Published:** 2025-05

**Authors:** Isla E. Emmen, William C. Vuyk, Andrew J. Lail, Sydney Wolf, Eli J. O’Connor, Rhea Dalvie, Maansi Bhasin, Aanya Virdi, Caroline White, Nura R. Hassan, Alex Richardson, Grace VanSleet, Andrea Weiler, Savannah Rounds-Dunn, Kenneth Van Horn, Marc Gartler, Jane Jorgenson, Michael Spelman, Sean Ottosen, Nicholas R. Minor, Nancy Wilson, Thomas C. Friedrich, David H. O’Connor

**Affiliations:** University of Wisconsin–Madison School of Medicine and Public Health, Madison, Wisconsin, USA (I.E. Emmen, W.C. Vuyk, A.J. Lail, S. Wolf, E.J. O’Connor, R. Dalvie, M. Bhasin, A. Virdi, C. White, N.R. Hassan, N. Wilson, D.H. O’Connor); Madison West High School, Madison (E.J O’Connor); University of Wisconsin–Madison, Wisconsin National Primate Research Center, Madison (A. Richardson, G. VanSleet, A. Weiler, T.C. Friedrich); Public Health Madison Dane County, Madison (S. Rounds-Dunn, K. Van Horn); Madison Public Library, Madison (M. Gartler, J. Jorgensen, M. Spelman, S. Ottosen); University of Wisconsin–Madison School of Veterinary Medicine, Madison (T.C. Friedrich)

**Keywords:** SARS-CoV-2, COVID-19, respiratory infections, viruses, severe acute respiratory syndrome coronavirus 2, SARS, coronavirus disease, coronavirus, public health, Wisconsin, RNA, United States

## Abstract

In the United States, SARS-CoV-2 genomic surveillance initially relied almost entirely on residual diagnostic specimens from nucleic acid amplification–based tests. However, use of those tests waned after the end of the COVID-19 Public Health Emergency on May 11, 2023. In Dane County, Wisconsin, we partnered with local- and state-level public health agencies and the South Central Library System to continue genomic surveillance by obtaining SARS-CoV-2 genome sequences from freely available community rapid antigen tests (RATs). During August 15, 2023–February 29, 2024, we received 227 RAT samples, from which we generated 127 sequences with >10× depth of coverage for >90% of the SARS-CoV-2 genome. In a subset of tests, lower cycle threshold values correlated with sequence success. Our results demonstrated that collecting and sequencing results from RATs in partnership with community sites is a practical approach for sustaining SARS-CoV-2 genomic surveillance.

Genomic surveillance is a powerful tool that can inform public health responses to disease outbreaks (*1*). During the COVID-19 pandemic, genomic surveillance data were used to identify variants of concern, investigate patterns of transmission, and develop effective vaccines (*2*–*4*).

Genomic surveillance requires large, representative sets of samples. Initially, nucleic acid amplification tests (NAATs) were the standard for detecting SARS-CoV-2 infection (*5*). Laboratories received residual nasal swab samples leftover from NAAT testing for viral sequencing through contracts with companies and clinics performing NAATs. After the COVID-19 public health emergency ended on May 11, 2023, NAAT testing in clinical and public health facilities declined precipitously as government subsidies for performing NAATs ended (*6*). During 2020, an average of 587,975 NAATs were performed weekly in the United States. By 2023, that number decreased to ≈96,215 tests per week (*7*). Subsequently, the primary source of samples for genomic surveillance was greatly diminished.

The US Food and Drug Administration issued the first emergency use authorization for a COVID-19 rapid antigen test (RAT) in August of 2020 (*8*). At-home RAT usage increased significantly in 2021 during the rise of the Omicron lineage (*9*). RATs are less expensive than NAATs, provide faster results, and do not require trained personnel (*10*). RATs usually involve swabbing the insides of both nostrils, placing the swab into an inactivation buffer, and applying the buffer onto a lateral flow test strip. If SARS-CoV-2 antigen is present, a colorimetric test line will indicate positivity (*11*). By July 2024, the United States had 38 available over-the-counter SARS-CoV-2 RAT products authorized by the Food and Drug Administration and available to the public (*12*).

Multiple groups have investigated RATs as source material for SARS-CoV-2 genomic surveillance. SARS-CoV-2 RNA can be recovered from antigen tests and sequenced (*13*–*17*), enabling tracking of circulating lineages. We hypothesized that community members would be willing to send in SARS-CoV-2–positive RATs for genomic surveillance if the process were sufficiently simple. Thus, we partnered with local public libraries and a public health agency to create and assess a system for persons to anonymously submit positive RATs for viral RNA analysis and sequencing. 

## Materials and Methods

### Collection of Rapid Antigen Tests

**Figure 1:**
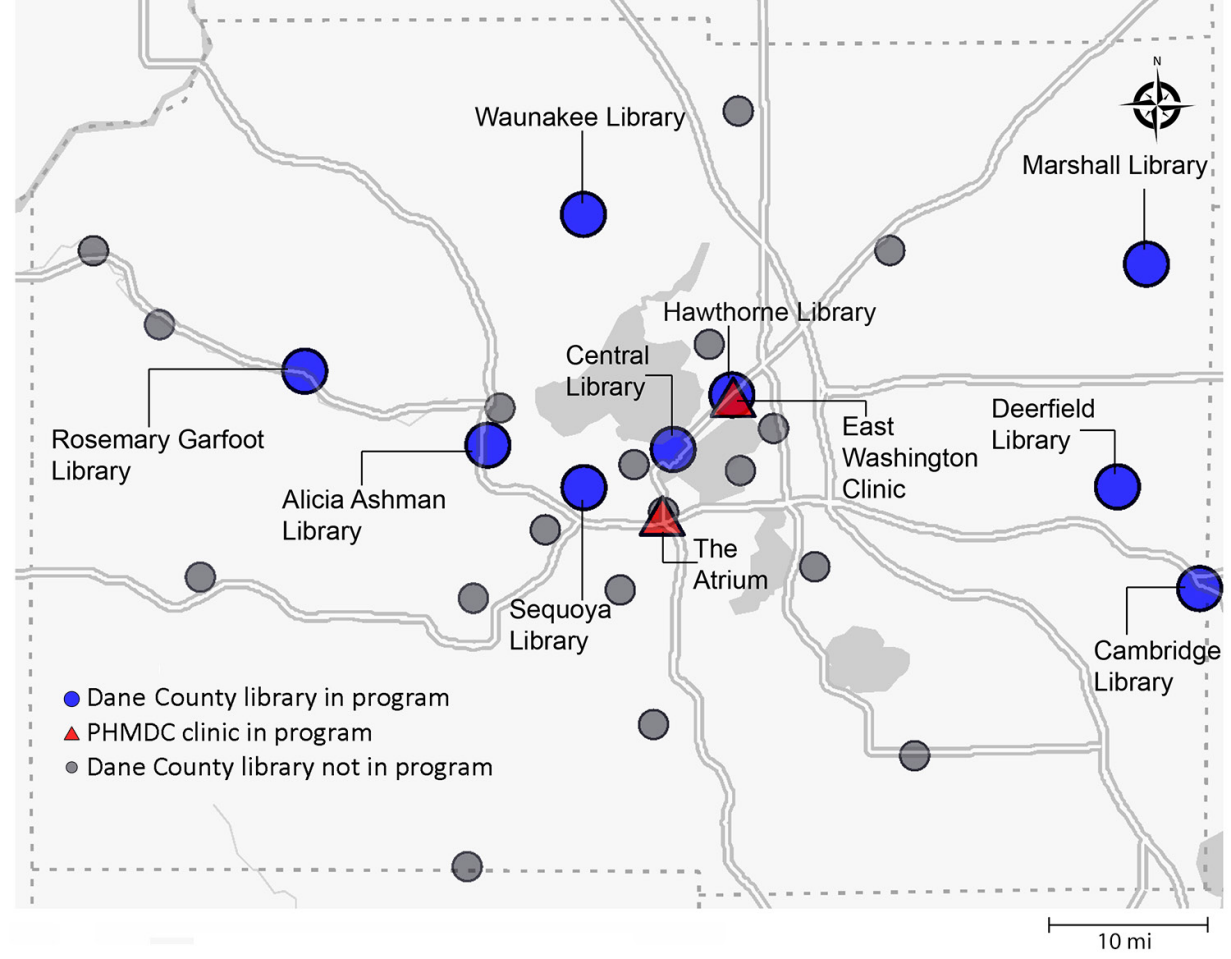
Locations for SARS-CoV-2 genomic surveillance from community-distributed rapid antigen tests, Wisconsin, USA. Nine of the South Central Library System libraries and 2 PHMDC sites distributed research packets and SARS-CoV-2 rapid antigen tests to patrons. Willing participants could send their positive tests to the AIDS Vaccine Research Laboratory, University of Wisconsin–Madison (Madison, WI, USA), for sequencing. PHMDC, Public Health Madison Dane County.

In Wisconsin, the South Central Library System and Public Health Madison Dane County (PHMDC) distributed RATs from the US national stockpile to the public free of charge. We partnered with 9 libraries and 2 sites through PHMDC. Six of the libraries were located in urban areas and 3 in rural areas (*18*) (Figure 1).

**Figure 2:**
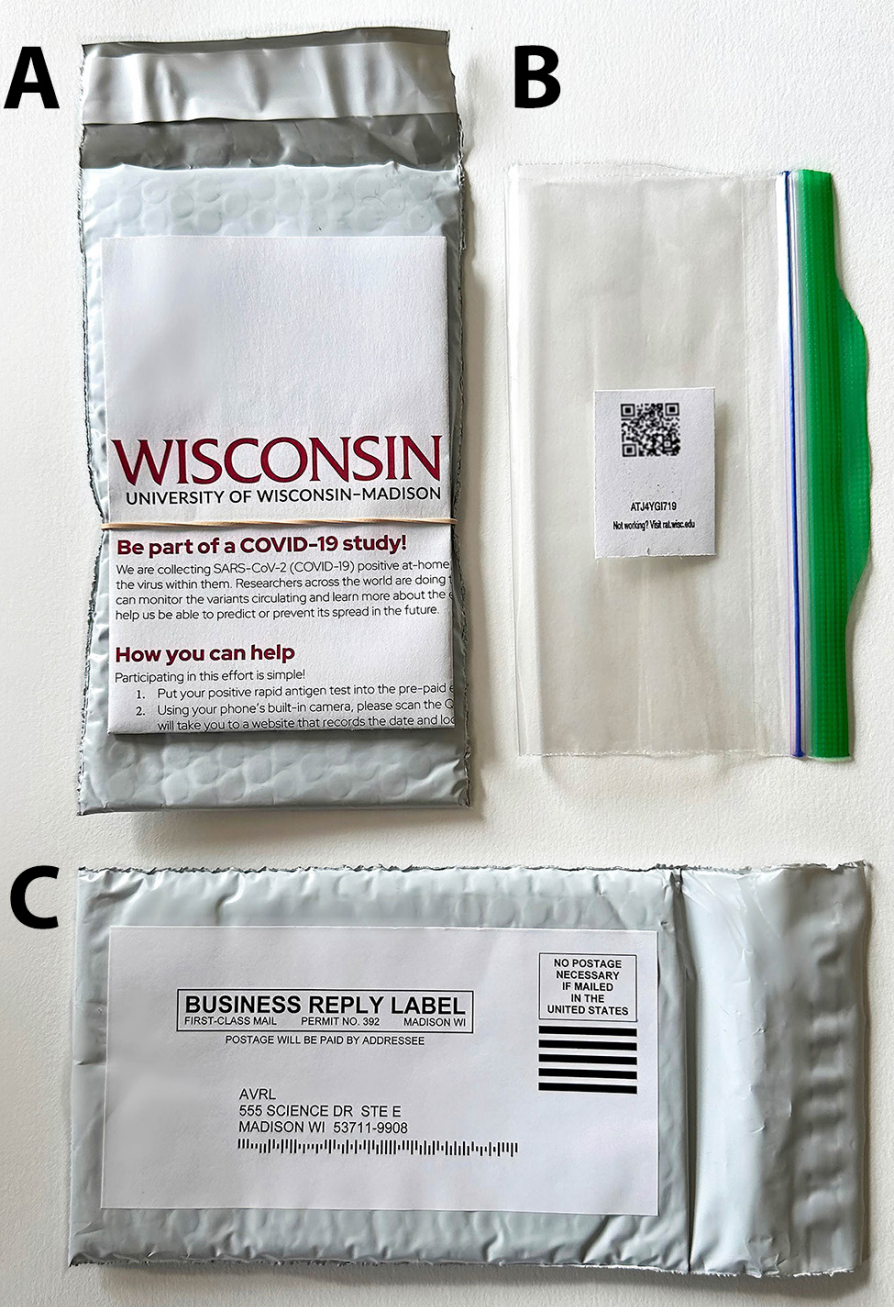
Research packets distributed for SARS-CoV-2 genomic surveillance from community-distributed rapid antigen tests, Wisconsin, USA. A) Envelope and instructions; B) zip-top bag included in packet with quick response (QR) code; C) return address label. Research packets were attached to SARS-CoV-2 rapid antigen test boxes, enabling participants to send their positive tests to the laboratory through the US Postal Service. A folded flyer (A), attached to an envelope, explained in both English and Spanish the goal of the study and how to participate. Participants scanned the QR code inside the included zip-top bag (B) to document the date and location of their rapid antigen test, then sealed their test strip inside. The location of the scanned QR code was immediately converted to the census block group of the scan and stored in a secure database. Participants returned test strips in the provided envelope, which had a business-reply shipping label (C), enabling participants to mail to our laboratory from any post office drop box.

We designed a packet of materials to attach to each RAT to enable collection of positive tests (Figure 2). The packet included an instructional flyer affixed to the outside of a bubble mailer to which a business reply mail shipping label was affixed. Inside the bubble mailer, we included a zip-top bag with a unique quick response (QR) barcode for return of RAT tests. The flyer had instructions in both English and Spanish, describing the study and providing instructions on how to participate (Appendix Figure).

Participants could volunteer to submit their RAT to our program if they tested SARS-CoV-2 positive. Participants were directed to scan the unique QR code on an internet-connected device, seal the positive RAT strip inside the zip-top bag, place the bag inside the bubble mailer and seal it, then drop the sealed mailer in any post office mailbox. The inactivation buffer in a RAT inactivates SARS-CoV-2, rendering the tests nonbiohazardous and safe to send through the mail (*19*).

Upon arrival at our laboratory, we scanned the QR code to record the date of receipt and stored at −80°C until processing. Most RATs we received were BinaxNOW COVID-19 Antigen Self Tests (Abbott, https://www.abbott.com) or iHealth COVID-19 Antigen Rapid Tests (iHealth Labs Inc., https://ihealthlabs.com).

### Ethics Statement

The University of Wisconsin institutional review board determined this project was human research exempt because participants were anonymous and self-identified. We created a secure website and database by using Node JS (https://nodejs.org) to collect the barcode, the date, and the location when a user scanned a randomly generated unique QR code. We assumed those data were a reasonable proxy for RAT date and location. The location of the scan was automatically converted to a census block group on the users’ machines before submission to our database, so the actual location of each submission was not known to the study team. A census block group contains 250­–550 housing units (*20*).

### Nucleic Acid Extraction

We developed our approach to extract nucleic acids from used RATs per previously describe methods (*13*). We thawed and opened RATs to retrieve the testing strip, which we placed into a clean 5-mL freezer tube (Sarstedt, https://www.sarstedt.com). Some RATs also included a nasal swab, and we also placed those in the freezer tube.

We added 800 μL of Viral Transport Medium (Rocky Mountain Biologicals, LLC, https://rmbio.com), and incubated the tube at room temperature for 10 minutes on a Hulamixer (Thermo Fisher Scientific, https://www.thermofisher.com). We transferred 500 μL of that mixture to a clean 1.5-mL tube and added 5 μL of Dynabeads Wastewater Virus Enrichment Beads (Thermo Fisher Scientific). We incubated samples for 10 minutes on a Hulamixer, then placed on a magnetic rack for 3 minutes. Once clear, we discarded the supernatant and resuspended the beads in 500 µL of lysis buffer. We returned the tube to the magnet for 3 minutes and then transferred the clear supernatant to a clean tube. We isolated samples on a Kingfisher Apex instrument (Thermo Fisher Scientific) following the manufacturer’s protocol (protocol no. MagMAX_Wastewater_DUO96.bdz).

After isolation, we treated the samples with Turbo DNase (Thermo Fisher Scientific), according to the manufacturer’s protocol. After DNase treatment, we cleaned samples by using the RNA Clean and Concentrator-5 kit (Zymo Research, https://www.zymoresearch.com), following the manufacturer’s protocol, but skipping the in-column DNase I Treatment.

### Quantitative Reverse Transcription PCR and Sequencing

We selected a random subset of 75 samples to investigate trends between the quantitative reverse transcription PCR cycle threshold (Ct) and sequencing quality. We quantified SARS-CoV-2 RNA using the CDC N1 Taqman assay (*21*) (Appendix).

We generated PCR amplicons by using the QIAseq DIRECT SARS-CoV-2 Kit with Booster and Enhancer (QIAGEN, https://www.qiagen.com), according to the manufacturer’s instructions. We normalized indexed samples to 4 nmol and pooled samples together. We diluted the pool to a concentration of 8 pmol and ran using 2 × 150 MiSeq Reagent Kits v2 on a MiSeq instrument (both Illumina, https://www.illumina.com).

### Sequencing Analysis

We quality-checked raw sequencing reads and aligned to the wild-type SARS-CoV-2 reference (GenBank accession no. MN908947.3), then variant-called by using the open-source viralrecon pipeline from the nf-core project (*22*,*23*; B.E. Langer et al., unpub. data, https://doi.org/10.1101/2024.05.10.592912). We set the minimum frequency threshold for variant-calling to 0.01. Further details for how we ran viralrecon, alongside the custom R scripts we used to generate figures, are available in our GitHub repository (https://github.com/dholab/Library-Rapid-Antigen-Test-Manuscript).

### Statistical Analysis

We used an unpaired 2-tailed *t*-test to compare the effect of Ct and length of transit time between samples that passed our sequencing quality threshold of >90% coverage at >10× depth and those that failed. We performed that analysis in Prism version 10.1.0 (GraphPad, https://www.graphpad.com).

We compared the identities of SARS-CoV-2 lineages detected in our RAT-derived sequences with surveillance data from the Wisconsin State Laboratory of Hygiene (WSLH) SARS-CoV-2 Genomic Dashboard (*24*). We analyzed data from August 28, 2023–February 25, 2024, dividing our passing sequences into 2-week intervals on the basis of test scan dates. We only included participant-scanned tests in that analysis. We assigned Pango lineages to our sequences by using Nextclade version 3.5.0 (*25*). For each 2-week period, we identified the 2 most prevalent lineage groups, which we based on Nextstrain clades, in the WSLH wastewater surveillance data and determined how often our RAT program also detected the same prevalent lineages.

## Results

### Test Collection

During August 15, 2023–February 29, 2024, we supplied 9 libraries and 2 public health clinics in Dane County with 7,775 research packets to attach to SARS-CoV-2 RATs distributed to patrons. Among distributed packets, 223 (2.9%) were mailed to our laboratory. Some packets contained multiple tests, resulting in 227 total tests for analysis. The return rates varied by month (Table 1), but the mean number received each month was 32 (SD 10).

**Table 1:** Monthly distribution and return of research packages in a study of SARS-CoV-2 genomic surveillance from community-distributed rapid antigen tests, Wisconsin, USA*

Collection month	Approximate no. packets supplied for RAT collection	No. packets with positive tests	No. tests that passed sequencing quality threshold†
2023			
Aug	100	13	5
Sep	1,470	33	14
Oct	1,390	37	18
Nov	2,405	33	21
Dec	300	46	28
2024			
Jan	1,160	28	20
Feb	950	33	21
Total no.	7,775	223	127

*RATs, rapid antigen tests. †Quality threshold was >10× depth of coverage for >90% of the SARS-CoV-2 genome.

Some tests arrived at the laboratory without the barcode or with a barcode that had never been scanned, resulting in loss of associated metadata. Of the 223 research packets received, 170 were properly associated with time and location metadata. Of those 170 samples, 1 was scanned in Sauk County, Wisconsin (adjacent to Dane County), and the rest were scanned in Dane County.

### Sequencing Quality

We sequenced SARS-CoV-2 from all 227 RATs. We considered a sequence with genome coverage >90% at a depth of coverage >10× to be a passing sequence. Of the 227 RAT-derived sequences, 128 (56%) passed (Appendix Table 1).

**Figure 3:**
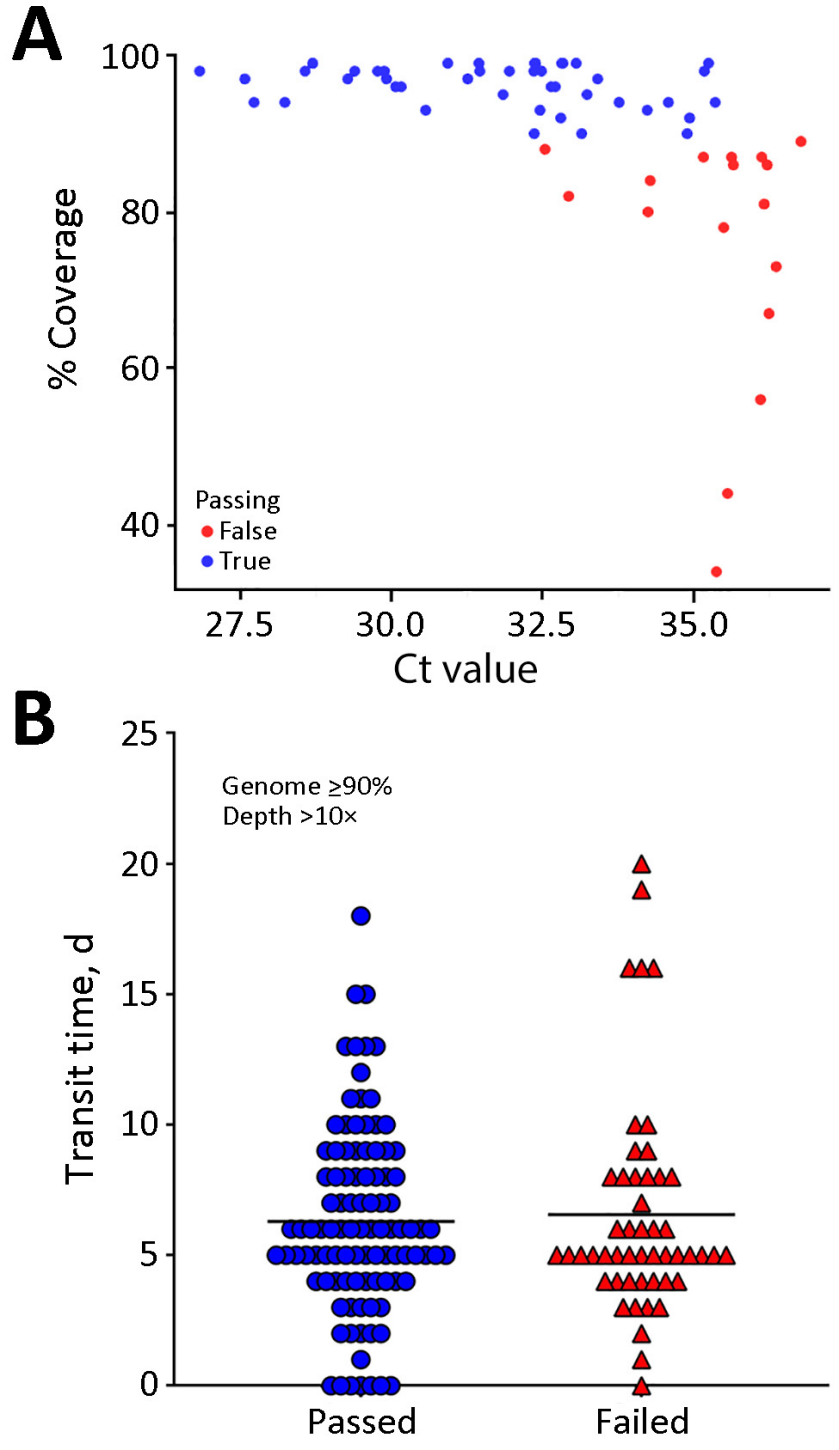
Comparison of passing and failing samples in a study of SARS-CoV-2 genomic surveillance from community-distributed rapid antigen tests, Wisconsin, USA. Scatterplots compare percentage coverage for Ct values (A) and transit times (B) for passing and failing RATs. Ct values were obtained through quantitative reverse transcription PCR. Sequences that passed the quality threshold had >90% coverage of the SARS-CoV-2 genome at >10× depth. The mean Ct for samples that passed was 31.7 and the mean Ct for those that failed was 35.3 (p<0.0001 by unpaired 2-tailed *t*-test; degrees of freedom = 59). Samples with lower Ct values correlated with higher SARS-CoV-2 coverage. Transit time refers to the number of days between a participant scanning the QR code provided with the RAT and receipt of positive RAT at our laboratory. The horizontal black line (B) is the mean value for each group. The mean transit time for passing samples was 6.3 (SD 3.6) days and the mean transit time for failing samples was 6.6 (SD 4.2) days. We noted no significant difference in transit times between passing and failing sequences (p = 0.69 by unpaired *t*-test). The amount of viral material present on RAT correlated with our ability to sequence samples, but time en route did not. Ct, cycle threshold; RAT, rapid antigen test.

Next, we evaluated whether SARS-CoV-2 viral RNA concentration or transit time correlated with successful sequencing. We randomly selected 75 samples for semiquantitative reverse transcription PCR. Of those samples, 15 had no detectable amplification of the N1 target. We obtained passing sequences for samples with Ct values up to 35.4. The mean Ct for samples that passed was 31.7 and the mean Ct for samples that failed was 35.3, a significant difference via unpaired, 2-tailed *t*-test (p<0.0001; degrees of freedom = 59) (Figure 3, panel A).

The time between a test being scanned by the participant and our receiving it (i.e., the transit time) ranged from 1 to 20 days, but transit time had little effect on sequencing success (Figure 3, panel B). The mean transit time for the passing samples was 6.3 days, compared with 6.6 days for failed samples (p = 0.69 by unpaired 2-tailed *t*-test).

### Tracking SARS-CoV-2 Lineages

**Figure 4:**
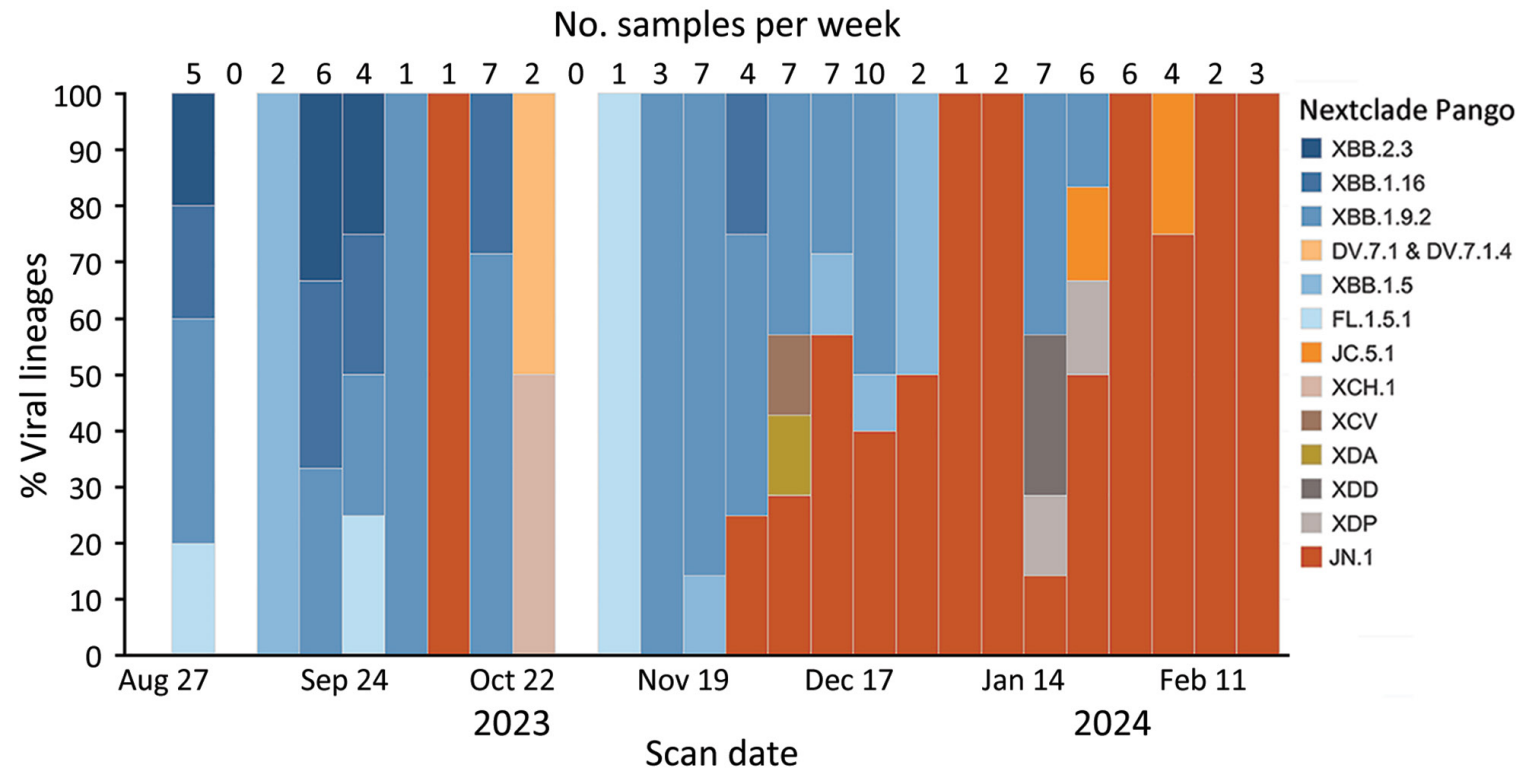
Number of samples collected per week and viral lineages detected in a study of SARS-CoV-2 genomic surveillance from community-distributed rapid antigen tests (RATs), Wisconsin, USA. The chart shows the percentage of SARS-CoV-2 lineages by week for samples that passed quality control thresholds of >90% of the SARS-CoV-2 genome at >10× depth. The date used reflected the date the participant scanned a provided QR code attached to a RAT. Unscanned RATs were excluded from the analysis. The number of samples included in each week’s percentage is shown above the bar. We assigned Pango lineages by using Nextclade version 3.5.0 (*25*). From August to mid-November 2023, the most common lineages in our samples fell under XBB.1.5, XBB.1.9.2, XBB.1.16, and XBB.2.3. Beginning in early December 2023, we began to see an increase in the number of samples belonging to the lineage JN.1, which dominated RAT samples scanned in February 2024.

We used Nextclade version 3.5.0 (*25*) to determine the Pango lineage of each successfully sequenced sample and tracked SARS-CoV-2 lineages detected by week on the basis of participant scan date (Figure 4). During August–November 2023, most detected lineages were assigned to the XBB clade. Beginning in December 2023, we observed a shift to the JN.1 lineage, which predominated in February 2024.

The identities of viral lineages in our RAT-derived sequences were concordant with statewide trends in lineages detected via wastewater surveillance, as summarized on the WSLH SARS-CoV-2 Wastewater Genomic Dashboard (*24*) (Table 2). Our program detected the dominant wastewater lineage in 12 of 13 two-week reporting periods and the second-most prevalent lineage in 7 of 13 periods. Concordance with wastewater surveillance data indicates that RAT-based surveillance can detect common circulating lineages. Moreover, RAT-based surveillance resulted in 6 of the earliest documented cases of a lineage in Wisconsin in GenBank and GISAID: JN.1.1, JN.1.2, XDD, XDA, XDP, and XDE (Table 3).

**Table 2:** Lineages detected during SARS-CoV-2 genomic surveillance from community-distributed rapid antigen tests, Wisconsin, USA*

Date†	Highest percentage‡			Second highest percentage§		No. RATs with passing sequence¶	Other lineages detected via RAT sequencing
Wastewater lineage	Matching RAT lineages	Wastewater lineage	Matching RAT lineages
2023 Aug 28	EG.5.1	EG.5.1.4		XBB.1.16	XBB.1.16.11	5	FL.1.5.1, XBB.2.3
2023 Sep 11	EG.5.1	EG.5.1.13		XBB.1.16	XBB.1.16	8	XBB.1.5.10, XBB.1.5, GE.1,HK.9, JF.1.1, HH.1
2023 Sep 25	EG.5.1	EG.5.1.4		XBB.1.16	XBB.1.16	4	GJ.1.2, FL.1.5.1
2023 Oct 9	EG.5.1	HV.1, HV.1.8		XBB.1.9	None	8	HK.29, JN.1.1, XBB.1.16.9
2023 Oct 23	EG.5.1	None		XBB.1.16	None	2	XCH.1, DV.7.1
2023 Nov 6	EG.5.1	EG.5.1.1, EG.5.1.6		XBB.1.16	None	6	FL.1.5.1, HK.13.2.1, HK.26
2023 No 20	EG.5.1	EG.5.1.1, EG.5.1.6, HV.1		XBB.1.16	XBB.1.16.6	10	HK.26, GK.1.1, JN.1.4.5, HK.3, JN.1
2023 Dec 4	EG.5.1	HV.1, HV.1.2, HV.1.6		BA.2.86	JN.1, JN.1.1, JN.1.38	14	XDA, XCV, GK.1.8
2023 Dec 18	BA.2.86	JN.1, JN.1.1, JN.1.4, JN.1.42		EG.5.1	EG.5.1, EG.5.1.8, HV.1	11	JG.3, GW.5.1.1, GK.1.6.1
2024 Jan 1	BA.2.86	JN.1, JN.1.39		EG.5.1	None	3	None
2024 Jan 15	BA.2.86	JN.1, JN.1.38, JN.1.4, JN.1.42		EG.5.1	HV.1	15	JG.3, XDD, XDP, JC.5.1, HK.3.2
2024 Jan 29	BA.2.86	JN.1, JN.1.1		EG.5.1	None	9	None
2024 Feb 12	BA.2.86	JN.1, JN.1.42		XBB.2.3	None	4	None

*Lineages represent successful rapid antigen test sequences that corresponded to the 2 most prevalent lineage groups in the wastewater signal in the state of Wisconsin for each 2-week period. RAT, rapid antigen test. †Dates represent first day of each 2-week reporting period. ‡Lineage group comprising the first largest percentage of wastewater data. §Lineage group comprising the second largest percentage of wastewater data. ¶Passing sequences had >90% genome coverage at >10× depth.

**Table 3:** Lineages detected in a study of SARS-CoV-2 genomic surveillance from community-distributed rapid antigen tests, Wisconsin, USA*

GenBank accession no.	Scanned test date	Pango lineage
PP761647	2023 Oct 14	JN.1.1
PP747716	2023 Dec 4	XDA
PP747739	2023 Dec 21	JN.1.2
PP747779	2023 Dec 22	XDE
PP747696	2024 Jan 17	XDD
PP747750	2024 Jan 24	XDP

*These samples were the earliest recorded examples of respective Pango lineage in Wisconsin according to data submitted to GISAID (https://www.gisaid.org) and GenBank as of April 18, 2024.

## Discussion

Genomic surveillance has been crucial for tracking SARS-CoV-2 evolution during the COVID-19 pandemic (*26*). Because most persons now use RATs instead of NAATs to diagnose SARS-CoV-2 infection, we sought to evaluate a community genomic surveillance program predicated on voluntary mailing of positive RATs.

Despite the common narrative that the public is disinterested in COVID-19, we observed surprisingly strong participation. During August 12, 2023–February 24, 2024, Dane County’s average COVID-19 test positivity rate, which we used to estimate the return rate on RATs, was 12.3% (range 7.8%–16%) (*27*). In an extreme case in which all the RATs distributed by our partners were used, we estimated that one quarter of all positive tests distributed with packets were returned to our laboratory for analysis. The true return rate is likely higher because some tests distributed with packets likely were not used.

The transit time during which RATs sat in uncontrolled (ambient) conditions had a negligible effect on overall sequencing success (Figure 3, panel B). Other studies have demonstrated that extraction of viral RNA is possible from RATs stored at room temperature for long periods (*14*,*16*); one study generated 75.2% genome coverage from a RAT stored at room temperature for 3 months. We obtained a sequence with >10× coverage for >90% of the SARS-CoV-2 genome from a RAT that sat at uncontrolled temperatures for at least 17 days. Taken together, those results highlight that RATs stored at uncontrolled temperatures can be mailed from the point-of-testing to centralized laboratories for sequencing. Most (98%) of the US population is served by the United States Postal Service (*28*). Thus, the ability to self-collect samples for mail-in analysis could enable genomic surveillance even in settings that are typically underserved by academic and clinical research.

SARS-CoV-2 lineages identified by our RAT surveillance program were similarly prevalent in Wisconsin’s statewide wastewater sequencing data (*24*). Of note, we also detected emerging lineages like JN.1 and rare variants like XDE, which was documented only 22 times in North America (*29*). Those findings demonstrate that RAT-based sequencing can effectively complement existing wastewater and NAAT surveillance methods.

One limitation of our study is the reliance on self-reported data, which is less precise than clinical specimen metadata. Our metadata depended on the participant’s QR code scan to approximate the date and location of the test, which might have reduced data accuracy. Another limitation is that ≈25% of packets arrived unscanned or without a barcode; thus, we had no metadata for those samples. Our only communication with participants was through the flyer provided with each packet, and some participants might only skim the instructions and misinterpret the protocol. To reduce the frequency of unscanned tests, more simplified instructions that include visual cues could more clearly communicate the directions for returning RATs.

Census block groups of scanned tests showed a strong bias toward urban locations (*18*); only 1 test was scanned by a participant in a rural census block group. The 3 packet distribution sites in rural areas of Dane County received only 3% of the total packets we supplied, which might partially account for that low number of tests from rural areas. Rural and underrepresented areas might need stronger engagement efforts in future studies to achieve more representative genomic surveillance.

Our program relied on freely available RATs provided from the national government stockpile. The long-term sustainability of the programs that distribute those tests is unknown, which means this system for collecting and sequencing RATs might not be sustainable long-term. A similar program could be established with RATs purchased by community members (e.g., by partnering with pharmacies to put them at point-of-sale), but that could greatly bias the results toward persons who have the resources and motivation to purchase costly tests. Providing free tests to members of the community gives them a valuable tool to minimize their risk for COVID-19 transmission while also potentially providing more inclusive, representative genomic surveillance.

In conclusion, the program described here could act as a framework for the creation of more expansive genomic surveillance programs. Regulators in some countries have approved at-home RATs for other respiratory viruses, including influenza A virus and respiratory syncytial virus (*30*–*32*), and those tests could be collected to set up surveillance programs for other viruses. Other studies have demonstrated the possibility of recovering various respiratory viruses from COVID-19 RATs (*15*,*33*). Thus, by collecting both positive and negative RATs from symptomatic persons, the prevalence of respiratory viruses circulating in communities could also be estimated, creating an innovative additional method for assessing the spread of respiratory viruses in communities.

This article was preprinted at https://www.medrxiv.org/content/10.1101/2024.08.12.24311680v1.

Appendix Additional information on SARS-CoV-2 genomic surveillance from community-distributed rapid antigen tests, Wisconsin, USA.

## References

[1] Ladner JT, Sahl JW. Towards a post-pandemic future for global pathogen genome sequencing. PLoS Biol. 2023;21:e3002225. 10.1371/journal.pbio.3002225 37527248 PMC10393143

[2] Rasmussen M, Møller FT, Gunalan V, Baig S, Bennedbæk M, Christiansen LE, et al. First cases of SARS-CoV-2 BA.2.86 in Denmark, 2023. Euro Surveill. 2023;28:36. 10.2807/1560-7917.ES.2023.28.36.2300460 37676147 PMC10486197

[3] Oliveira Roster KI, Kissler SM, Omoregie E, Wang JC, Amin H, Di Lonardo S, et al. Surveillance strategies for the detection of new pathogen variants across epidemiological contexts. PLOS Comput Biol. 2024;20:e1012416. 10.1371/journal.pcbi.1012416 39236073 PMC11407617

[4] Robishaw JD, Alter SM, Solano JJ, Shih RD, DeMets DL, Maki DG, et al. Genomic surveillance to combat COVID-19: challenges and opportunities. Lancet Microbe. 2021;2:e481–4. 10.1016/S2666-5247(21)00121-X 34337584 PMC8315763

[5] World Health Organization. Recommendations for national SARS-CoV-2 testing strategies and diagnostic capacities [cited 2024 Apr 29]. https://www.who.int/publications/i/item/WHO-2019-nCoV-lab-testing-2021.1-eng

[6] Kates J, Cubanski J, Cox C, Published JT. Timeline of end dates for key health-related flexibilities provided through COVID-19 emergency declarations, legislation, and administrative actions [cited 2024 Nov 20]. https://www.kff.org/coronavirus-covid-19/issue-brief/timeline-of-end-dates-for-key-health-related-flexibilities-provided-through-covid-19-emergency-declarations-legislation-and-administrative-actions

[7] Centers for Disease Control and Prevention. COVID data tracker [cited 2024 May 9]. https://covid.cdc.gov/covid-data-tracker

[8] Centers for Disease Control and Prevention. COVID Museum COVID-19 timeline [cited 2025 Mar 6]. https://www.cdc.gov/museum/timeline/covid19.html

[9] Rader B, Gertz A, Iuliano AD, Gilmer M, Wronski L, Astley CM, et al. Use of at-home COVID-19 tests—United States, August 23, 2021–March 12, 2022. MMWR Morb Mortal Wkly Rep. 2022;71:489–94. 10.15585/mmwr.mm7113e1 35358168 PMC8979595

[10] Khalid MF, Selvam K, Jeffry AJN, Salmi MF, Najib MA, Norhayati MN, et al. Performance of rapid antigen tests for COVID-19 diagnosis: a systematic review and meta-analysis. Diagnostics (Basel). 2022;12:110. 10.3390/diagnostics12010110 35054277 PMC8774565

[11] American Society for Microbiology. How the SARS-CoV-2 EUA antigen tests work [cited 2024 Jul 25]. https://asm.org:443/Articles/2020/August/How-the-SARS-CoV-2-EUA-Antigen-Tests-Work

[12] Food and Drug Administration. At-home OTC COVID-19 diagnostic tests [cited 2024 Jul 16]. https://www.fda.gov/medical-devices/coronavirus-covid-19-and-medical-devices/home-otc-covid-19-diagnostic-tests

[13] Martin GE, Taiaroa G, Taouk ML, Savic I, O’Keefe J, Quach R, et al. Maintaining genomic surveillance using whole-genome sequencing of SARS-CoV-2 from rapid antigen test devices. Lancet Infect Dis. 2022;22:1417–8. 10.1016/S1473-3099(22)00512-6 35934015 PMC9352270

[14] Rector A, Bloemen M, Schiettekatte G, Maes P, Van Ranst M, Wollants E. Sequencing directly from antigen-detection rapid diagnostic tests in Belgium, 2022: a gamechanger in genomic surveillance? Euro Surveill. 2023;28:91. 10.2807/1560-7917.ES.2023.28.9.2200618 36862099 PMC9983067

[15] Paull JS, Petros BA, Brock-Fisher TM, Jalbert SA, Selser VM, Messer KS, et al. Optimisation and evaluation of viral genomic sequencing of SARS-CoV-2 rapid diagnostic tests: a laboratory and cohort-based study. Lancet Microbe. 2024;5:e468–77. 10.1016/S2666-5247(23)00399-3 38621394 PMC11322816

[16] Nguyen PV, Carmola LR, Wang E, Bassit L, Rao A, Greenleaf M, et al. SARS-CoV-2 molecular testing and whole genome sequencing following RNA recovery from used BinaxNOW COVID-19 antigen self tests. J Clin Virol. 2023;162:105426. 10.1016/j.jcv.2023.105426 37028004 PMC10036152

[17] Macori G, Russell T, Barry G, McCarthy SC, Koolman L, Wall P, et al. Inactivation and recovery of high quality RNA from positive SARS-CoV-2 rapid antigen tests suitable for whole virus genome sequencing. Front Public Health. 2022;10:863862. 10.3389/fpubh.2022.863862 35592078 PMC9113430

[18] Health Innovation Program. ZIP codes by rural and urban groupings: HIPxChange [cited 2025 Jan 3]. https://hipxchange.org/toolkit/ruralurbangroups

[19] Coelho FF, da Silva MA, Lopes TB, Polatto JM, de Castro NS, Andrade LAF, et al. SARS-CoV-2 rapid antigen test based on a new anti-nucleocapsid protein monoclonal antibody: development and real-time validation. Microorganisms. 2023;11:2422. 10.3390/microorganisms11102422 37894080 PMC10608853

[20] US Census Bureau. Geographic areas reference manual. Washington: the Bureau; 1994.

[21] Lu X, Wang L, Sakthivel SK, Whitaker B, Murray J, Kamili S, et al. US CDC real-time reverse transcription PCR panel for detection of severe acute respiratory syndrome coronavirus 2. Emerg Infect Dis. 2020;26:1654–65. 10.3201/eid2608.201246 32396505 PMC7392423

[22] Patel H, Monzón S, Varona S, Espinosa-Carrasco J, Garcia MU, Heuer ML, et al. nf-core/viralrecon: nf-core/viralrecon v2.6.0–rhodium raccoon [cited 2024 May 29]. https://zenodo.org/record/7764938

[23] Ewels PA, Peltzer A, Fillinger S, Patel H, Alneberg J, Wilm A, et al. The nf-core framework for community-curated bioinformatics pipelines. Nat Biotechnol. 2020;38:276–8. 10.1038/s41587-020-0439-x 32055031

[24] Wisconsin State Laboratory of Hygiene. SARS-CoV-2 wastewater genomic dashboard [cited 2024 Jun 7]. https://dataportal.slh.wisc.edu/sc2-ww-dashboard

[25] Aksamentov I, Roemer C, Hodcroft E, Neher R. Nextclade: clade assignment, mutation calling and quality control for viral genomes. J Open Source Softw. 2021;6:3773. 10.21105/joss.03773

[26] Tosta S, Moreno K, Schuab G, Fonseca V, Segovia FMC, Kashima S, et al. Global SARS-CoV-2 genomic surveillance: What we have learned (so far). Infect Genet Evol. 2023;108:105405. 10.1016/j.meegid.2023.105405 36681102 PMC9847326

[27] Public Health Madison & Dane County. Respiratory illness dashboard [cited 2024 Jun 7]. https://publichealthmdc.com/health-services/respiratory-illness/dashboard

[28] US Postal Service. Postal Service delivery performance continues to average 2.6 days [cited 2024 Nov 13]. https://about.usps.com/newsroom/national-releases/2023/1222-usps-delivery-performance-continues-to-average-2-6-days.htm

[29] Chen C, Nadeau S, Yared M, Voinov P, Xie N, Roemer C, et al. CoV-Spectrum: analysis of globally shared SARS-CoV-2 data to identify and characterize new variants. Bioinformatics. 2022;38:1735–7. 10.1093/bioinformatics/btab856 34954792 PMC8896605

[30] Food and Drug Administration. Influenza diagnostic tests [cited 2024 Aug 2]. https://www.fda.gov/medical-devices/in-vitro-diagnostics/influenza-diagnostic-tests

[31] Therapeutic Goods Administration. Respiratory combo panel RSV/SARS-CoV-2/Influenza A/B Rapid Antigen Test Kit RAT-19 (self-test) (nasal swab) (combination self‐tests) [cited 2024 Nov 14]. https://www.tga.gov.au/resources/covid-19-test-kits/respiratory-combo-panel-rsv-sars-cov-2-influenza-ab-rapid-antigen-test-kit-rat-19-self-test-nasal-swab-combination-self-tests

[32] Therapeutic Goods Administration. COVID-19, Influenza A/B & RSV Antigen Nasal Test Kit for self-testing (Biolink Biopen) [cited 2024 Nov 14]. https://www.tga.gov.au/resources/covid-19-test-kits/covid-19-influenza-ab-rsv-antigen-nasal-test-kit-self-testing-biolink-biopen

[33] Smith-Jeffcoat SE, Mellis AM, Grijalva CG, Talbot HK, Schmitz J, Lutrick K, et al.; RVTN-Sentinel Study Group. SARS-CoV-2 viral shedding and rapid antigen test performance— respiratory virus transmission network, November 2022–May 2023. MMWR Morb Mortal Wkly Rep. 2024;73:365–71. 10.15585/mmwr.mm7316a2 38668391 PMC11065460

