# Supplementary material for: SARS-CoV-2 Genomic Surveillance from Community-Distributed Rapid Antigen Tests, Wisconsin, USA
Source: Emerg Infect Dis. 2025 May;31(Suppl 1):S61–9. doi: 10.3201/eid3113.241192 (PMC12078534; doi:10.3201/eid3113.241192)
Supplement: Appendix — Additional information on SARS-CoV-2 genomic surveillance from community-distributed rapid antigen tests, Wisconsin, USA. [file 24-1192-Techapp-s1.pdf]

Article DOI: https://doi.org/10.3201/eid3113.241192

EID cannot ensure accessibility for supplementary materials supplied by authors. Readers who have difficulty accessing supplementary content should contact the authors for assistance.

SARS-CoV-2 Genomic Surveillance from Community-Distributed Rapid Antigen Tests, Wisconsin, USA

Appendix

Additional Methods

To quantify SARS-CoV-2 RNA, we used the CDC N1 Taqman assay (*1*). The forward primer sequence was "GACCCCAAAATCAGCGAAAT," the reverse primer sequence was "TCTGGTTACTGCCAGTTGAATCTG," and the probe sequence was "ACCCCGCATTACGTTTGGTGGACC." A 20 uL reaction was set up with 5 uL of Taqman Fast Virus 1-step Master Mix (ThermoFisher Scientific, https://www.thermofisher.com), 1.5 uL of SARS-COV2 RUO Primer/probe kit N1 (Integrated DNA Technologies, https://sg.idtdna.com), 8.5 uL of nuclease-free water, and 5 uL of sample RNA. The assay was run on a LightCycler 96 instrument (Roche, https://www.roche.com) with cycling conditions of 37°C for 2 min, 50°C for 15 minutes, 95°C for 2 minutes, and 50 cycles of 95°C for 3 seconds and 55°C for 30 seconds.

To confirm successful isolation of viral nucleic acid from the RAT substrate, each sample was also tested for the presence of human ribonuclease P nucleic acid. Only samples which tested positive for ribonuclease P were included in the analysis.

Reference

1. Lu X, Wang L, Sakthivel SK, Whitaker B, Murray J, Kamili S, et al. US CDC real-time reverse transcription PCR panel for detection of severe acute respiratory syndrome coronavirus 2. Emerg Infect Dis. 2020;26:1654–65. PubMed https://doi.org/10.3201/eid2608.201246

Appendix Table 1. Sequencing metrics for each rapid antigen test sample

Sample Name	% Coverage >10×	Input reads	Mapped reads
Lib RAT 1 CC	89	292,498	149451
Lib RAT 2 CC	99	429,670	379054
Lib RAT 3 CC	87	906,378	386605
Lib RAT 4 CC	99	751,628	560350
Lib RAT 5 CC	96	638,864	509354
Lib RAT 6 CC	73	939,016	248200
Lib RAT 7 CC	0	0	0
Lib RAT 8 CC	38	890,258	246596
Lib RAT 9 CC	0	2	0
Lib RAT 10 CC	97	576,444	363896
Lib RAT 11 CC	26	1,031,716	216466
Lib RAT 12 CC	69	739,654	296681
ATQC4UXFHL	94	567,612	469708
ATGRM0X3NG	99	518,862	475772
AT45CHNPMD	89	814,054	306016
ATFFPBDGC7	99	901,902	587391
ATRUPA5DJ5	97	644,592	553230
ATKCPI0YMU	98	121,384	115142
ATU2UEZGUU	91	920,378	687997
AT25TA545F	95	569,574	516194
ATAC3S9AX4	99	463,772	426031
AT1XJPLMT1	50	1,835,618	389978
ATX2RLNE3M	99	508,792	451110
AT9M50GXL5	98	565,532	380139
ATFY3C92IS	0	506	82
ATBUK9BEKQ	46	703,636	118219
AT1REAXVVZ	91	720,132	354056
ATHXJM6OTS	99	479,808	413933
No barcode 1	0	788	734
AT7V74R0RW	99	412,494	341780
ATXPYXN5EQ	88	842,312	258262
ATC50LYO64	5	1,743,894	369998
ATILJWETL2	0	700	622
ATGCWPGJ2V	9	1,224	1116
AT7ZH839N8	94	1,170,808	226765
ATYFNYVCLA	26	1,772	1622
AT2I45F27V	0	960	862
ATHDDZ3Z8S	83	830,390	295767
AT13LPB6FO	46	1,318,052	214090
ATL5VRS761	48	883,866	154114
ATZB3XY1WP	79	722,400	172355
AT137YUSYR	0	1,022	914
ATF5O133LQ	96	585,228	198721
ATRBJD97CM	100	2,942,536	2511305
AT6DRTU76H	37	843,298	133620
ATUQ7230RN	99	991,114	276971
AT5UTIINHL	98	764,184	171129
AT0MKPPLPR	68	668,400	132895
ATCLJ5EIQG	99	448,282	317652
ATPCXFPKEX	59	765,374	120615
AT8X5RUNJL	97	484,554	290255
AT5R2HNUD3	57	1,295,960	196832
AT2HXD2MB5	1	1,142,774	151226
AT4CHNRA3T	3	930,698	101019
ATCOXC4GN2	8	1,405,954	226976
AT06HSAZ8J	99	409,808	356320
ATWGJ53DEL	0	611,014	85770
AT0TFNDWZG	93	350,110	203864
AT6PMLSMAT	71	677,646	118692
ATBSEDOKCN	93	452,264	293360
ATAU1LDLE2	0	624,690	93593
AT4FE9C8N9	84	577,310	128734
ATFY0YNZ33	84	686,796	154397
ATNGW65XYZ	86	414,068	315045
AT3K7JLNGT	14	758,206	118737
ATP3ZOJFI9	1	1,039,286	123049
ATM6YA1R0Z	99	507,290	288831

Sample Name	% Coverage >10×	Input reads	Mapped reads
No_barcode_13	81	890,958	246545
ATNTXE6UZQ	98	824,780	384688
ATUZ85D2VU	99	682,910	494599
AT1W159O34	32	563,916	109791
ATP8WMDMZK	100	858,962	701834
ATAJAL9JG4	98	654,744	458966
ATCZTKHDF4	100	783,938	658101
ATP2AGQNK1	77	517,578	308320
ATTYEJSDWY	100	462,752	374615
ATSMWG98PK	100	293,600	252912
ATCRC6FGN5	0	2	0
ATG0HMTYYE	99	528,428	372524
No barcode 2	93	851,948	300344
ATGLSXIQIT	0	2	0
ATBH4IK27M	99	460,436	284508
ATA5WLTAK0	97	388,442	338390
ATV6AW7859	99	530,482	424853
ATACET5NJ8	99	686,048	262110
ATR9JWB9VK	0	0	0
ATMYVE1QEK	96	601,906	213699
ATNB4UEUAK	98	691,016	475180
ATKR3E8K57	99	366,958	304051
ATEHBAOC25	100	358,936	309877
ATG75FDZ90	97	345,068	270151
ATXXSAVO9F	99	454,890	386104
ATRX649RNA	98	349,086	148045
ATS90Q5O1U	85	612,284	128288
ATBW04JJJ9	99	415,074	343636
AT5M1NUPQS	99	369,402	309504
ATWY6KKEA5	63	616,968	112531
ATQN1DOYOX	95	366,642	285513
ATJUCHFV8Z	99	518,570	295505
ATZYNED0C2	99	492,508	394212
ATCVEU14QN	80	785,162	155348
ATZBGZQ7GK	42	553,134	98761
ATALAP7D6L	91	525,094	211252
No barcode 4	97	559,098	354832
ATFDG7UFZM	85	468,376	139461
ATTVOS73K4	99	494,720	395135
AT9E5WUVE3	99	607,072	307272
AT0U00ZFF4	59	761,988	160234
ATPDK4X5OH	94	398,224	312593
ATTYI7D5JH	92	317,232	224871
AT8DP3WGHB	95	373,098	308633
ATHLVVS4Y4	0	753,466	124774
ATM53BCTFV	93	523,742	202461
ATLOX1CQAQ	79	544,806	175590
ATPVOTRGYM	99	172,966	146251
AT0RH4DFUV	93	573,020	480617
ATZ0NRXDCK	95	206,960	178578
ATGUE1WYC7	53	493,418	176869
ATCZIZF68Z	74	582,974	176985
ATNTDGQ0ZV	96	306,770	254238
ATBZPSGVAB	99	329,430	291826
ATJ0KWF0TS	98	378,290	168951
ATWJ2D0980	98	382,652	301589
ATMDAY79PQ	86	513,622	260852
ATCXJM1DBO	96	419,218	289,501
ATKJWPMFO2	76	403,958	204,195
NO BARCODE "TIZZY"	92	178,138	162,858
ATHJWSM3HA	78	508,560	157,250
AT3ZC8GJPW	99	344,554	306,681
ATFGKWPAZL	95	383,338	319,075
ATFX9LZCHR	99	357,588	288,248
AT4VBM7WQH	97	490,514	377,684
AT96YQ9U13	99	382,846	316,223
AT4J8ARPEG	99	266,736	228,946
No Barcode 6	81	562,934	273,771

Sample Name	% Coverage >10×	Input reads	Mapped reads
ATJR9OSFWD	99	651,960	287,733
AT7FWDGTYF	55	717,400	190,486
ATWYRUEAD7	47	628,916	126,046
ATPLL5N312	67	577,476	194,399
AT7MY3T1E9	0	0	0
No Barcode 5 BN	95	536,304	384,937
No Barcode 5 ihealth	99	421,328	365,889
ATUKMG6K7J	98	463,306	295,424
ATZBGZQ7GK-2	10	782,986	84,241
ATPXOOQ032	91	400,474	296,893
ATPL7GXV96	76	891,358	275,845
ATPWR5IE4D	100	433,216	384,950
ATZ8EZY7IR	94	550,374	299,563
AT1CHWU504	5	995,852	159,976
ATQOATMEB2	69	669,062	87,444
No BARCODE 7	96	234,600	207,717
ATEZEING5R	98	525,892	470,022
ATPAVSLTVZ	98	515,332	447,870
AT0ERFB1HF	99	401,720	346,009
ATSAO2XRUQ-2	98	608,670	532,227
ATZJS41EVX	98	574,852	337,597
AT781XIB54	98	669,908	541,534
ATPDSVRWH9	97	527,216	455,656
AT0AWTMMKP	96	492,222	428,600
ATSAO2XRUQ	96	479,342	351,492
ATOI9WCZ81	99	743,776	505,064
ATDDTZIOII	97	680,874	513,429
ATO4IQDBLG	99	519,930	423,851
AT76LFJKEJ	95	724,068	544,041
AT7LKS02ZY	98	661,996	504,998
ATMBVXFJQN	98	514,408	403,974
ATHVKKGPO2	99	576,912	313,256
ATHX4ESJMU	99	363,530	209,804
AT4G966SRR	98	727,058	580,634
AT4J6GKZJV	96	642,310	347,757
AT4C6YKBZZ	96	554,572	387,180
ATOMINHMVO	99	641,806	262,231
AT7H83A7O2	90	586,554	283,807
ATRVT2XAYT	95	682,452	469,014
ATODO9Q9H0	95	486,940	292,397
AT05KGLKQ6	93	618,334	226,536
ATN3EBDCSD	92	610,662	259,921
ATWXMLXGNP	99	798,352	300,537
ATJ92HKIPP	44	961,196	207,289
ATBSWKRXY3	86	948,954	321,611
ATEEO64Q5W	87	688,572	253,601
AT25WIBJFT	94	938,212	255,607
AT5QBZRJ2P	86	750,942	337,419
ATKOAUDDAH-2	99	517,210	337,659
ATU0HRPQER	56	569,040	99,157
ATUB1RTN5L	97	430,372	377,749
ATXI9FLPF9	34	873,854	167,279
AT32O2dczr	0	10	2
AT5CGJV0G0	0	58	16
Ath33n84qi	0	6	4
ATKC3UWIMM	0	22	2
ATKOAUDDAH	0	8	0
ATXJTJUN2K	0	54	44
ATZPCYXSJB	0	22	14
No_barcode_14	94	636,822	369,131
No barcode 8	97	760,660	370,204
No barcode 9	80	183,516	51,627
30147_AT0KKXXXTO_01	82	1,352,878	336,231
30147_AT1F559LBQ_01	81	1,046,026	333,562
30147_AT2T3FKNIS_01	98	812,156	673,725
30147_AT61VLINZO_01	71	922,942	277,725
30147_AT67EY0KZD_01	73	950,004	230,564
30147_AT7M9SP0UD_01	94	840,602	633,794

Sample Name	% Coverage >10×	Input reads	Mapped reads
30147_AT85DBPO8X_01	89	976,366	405,103
30147_ATEJ3VUYVU_01	94	440,728	393,406
30147_ATF1KO758R_01	87	908,078	251,562
30147_ATH9QR4BYN_01	97	729,502	645,676
30147_ATIHCP9JIH_01	93	705,846	613,468
30147_ATKKB24DOT_01	88	516,452	445,004
30147_ATLMMUI9VT_01	68	942,500	204,324
30147_ATM2N85W29_01	90	1,166,916	683,261
30147_ATMRYCFWRL_01	94	1,069,858	592,132
30147_ATN7ZZTTSN_01	90	509,106	281,025
30147_ATPHZDSO0Y_01	87	868,694	239,250
30147_ATR1EVNFS3_01	82	1,009,044	739,865
30147_ATRGK0DY0P_01	44	1,218,360	224,435
30147_ATTG78KYMH_01	76	1,161,658	410,103
30147_ATTKH7F78W_01	67	1,018,114	269,172
30147_ATTX5ZVWSC_01	2	1,396,058	243,182
30147_ATVRGVTXY7_01	4	1,325,366	253,608
30147_ATWB8820OC_01	93	617,134	535,170
30147_ATWI85964T_01	78	1,351,490	296,960
30147_ATXQQQFFSL_01	84	672,456	571,298
30147_ATXY5LQ12H_01	99	977,534	461,780
30147_ATY86E0TCY_01	88	1,321,354	319,201
30147_ATYIMJ1U3N_01	98	577,478	208,854
30147_ATZ2JDFJD1_01	92	627,986	294,178

Appendix Table 2. List of accession numbers for GenBank and SRA and virus names for GISAID to access the rapid antigen test sequences generated as part of our library rapid antigen test community surveillance program

Internal ID	SRA accession no.	GenBank accession no.	GISAID virus name
ATX2RLNE3M_2	SAMN40752681	PP761650	hCoV-19/USA/WI-UW-15394/2023
ATAC3S9AX4_2	SAMN40752680	PP761651	hCoV-19/USA/WI-UW-15393/2023
AT25TA545F_2	SAMN40752679	PP761655	hCoV-19/USA/WI-UW-15392/2023
ATKCPI0YMU_2	SAMN40752678	PP761653	hCoV-19/USA/WI-UW-15391/2023
ATRUPA5DJ5_2	SAMN40752677		hCoV-19/USA/WI-UW-15390/2023
ATGRM0X3NG_2	SAMN40752676	PP761657	hCoV-19/USA/WI-UW-15389/2023
ATQC4UXFHL_2	SAMN40752675	PP761658	hCoV-19/USA/WI-UW-15388/2023
ATX2RLNE3M_1	SAMN40752674	PP761649	hCoV-19/USA/WI-UW-15387/2023
ATAC3S9AX4_1	SAMN40752673	PP761647	hCoV-19/USA/WI-UW-15386/2023
AT25TA545F_1	SAMN40752672	PP761652	hCoV-19/USA/WI-UW-15385/2023
ATKCPI0YMU_1	SAMN40752671	PP761659	hCoV-19/USA/WI-UW-15384/2023
ATRUPA5DJ5_1	SAMN40752670		hCoV-19/USA/WI-UW-15383/2023
ATFFPBDGC7_1	SAMN40752669	PP761648	hCoV-19/USA/WI-UW-15382/2023
ATGRM0X3NG_1	SAMN40752668	PP761654	hCoV-19/USA/WI-UW-15381/2023
ATQC4UXFHL_1	SAMN40752667	PP761656	hCoV-19/USA/WI-UW-15380/2023
ATM6YA1R0Z	SAMN40752666	PP747776	hCoV-19/USA/WI-UW-15517/2023
No-barcode-8	SAMN40752665	PP747769	hCoV-19/USA/WI-UW-15516/2024
No-barcode-7	SAMN40752664	PP747798	hCoV-19/USA/WI-UW-15515/2024
ATWXMLXGNP	SAMN40752663	PP747708	hCoV-19/USA/WI-UW-15514/2024
ATUB1RTN5L	SAMN40752662	PP747774	hCoV-19/USA/WI-UW-15513/2024
ATSAO2XRUQ-2	SAMN40752661	PP747705	hCoV-19/USA/WI-UW-15512/2024
ATSAO2XRUQ-1	SAMN40752660	PP747696	hCoV-19/USA/WI-UW-15511/2024
ATRVT2XAYT	SAMN40752659	PP747764	hCoV-19/USA/WI-UW-15510/2024
ATPDSVRWH9	SAMN40752658	PP747781	hCoV-19/USA/WI-UW-15509/2024
ATPAVSLTVZ	SAMN40752657	PP747743	hCoV-19/USA/WI-UW-15508/2024
ATOMINHMVO	SAMN40752656	PP747702	hCoV-19/USA/WI-UW-15507/2024
ATOI9WCZ81	SAMN40752655	PP747775	hCoV-19/USA/WI-UW-15506/2024
ATODO9Q9H0	SAMN40752654	PP747730	hCoV-19/USA/WI-UW-15505/2024
ATO4IQDBLG	SAMN40752653	PP747734	hCoV-19/USA/WI-UW-15504/2024
ATN3EBDCSD	SAMN40752652	PP747733	hCoV-19/USA/WI-UW-15503/2024
ATMBVXFJQN	SAMN40752651	PP747750	hCoV-19/USA/WI-UW-15502/2024
ATKOAUDDAH-2	SAMN40752650	PP747765	hCoV-19/USA/WI-UW-15501/2024
ATHX4ESJMU	SAMN40752649	PP747738	hCoV-19/USA/WI-UW-15500/2024
ATHVKKGPO2	SAMN40752648	PP747700	hCoV-19/USA/WI-UW-15499/2024
ATEZEING5R	SAMN40752647	PP747744	hCoV-19/USA/WI-UW-15498/2024
ATDDTZIOII	SAMN40752646	PP747740	hCoV-19/USA/WI-UW-15497/2024
AT7LKS02ZY	SAMN40752645	PP747770	hCoV-19/USA/WI-UW-15496/2024
AT7H83A7O2	SAMN40752644	PP747711	hCoV-19/USA/WI-UW-15495/2024

Internal ID	SRA accession no.	GenBank accession no.	GISAID virus name
AT781XIB54	SAMN40752643	PP747701	hCoV-19/USA/WI-UW-15494/2024
AT76LFJKEJ	SAMN40752642	PP747723	hCoV-19/USA/WI-UW-15493/2024
AT4J6GKZJV	SAMN40752641	PP747693	hCoV-19/USA/WI-UW-15492/2024
AT4G966SRR	SAMN40752640	PP747749	hCoV-19/USA/WI-UW-15491/2024
AT4C6YKBZZ	SAMN40752639	PP747793	hCoV-19/USA/WI-UW-15490/2024
AT25WIBJFT	SAMN40752638	PP747697	hCoV-19/USA/WI-UW-15489/2024
AT0ERFB1HF	SAMN40752637	PP747698	hCoV-19/USA/WI-UW-15488/2024
AT0AWTMMKP	SAMN40752636	PP747760	hCoV-19/USA/WI-UW-15487/2024
AT05KGLKQ6	SAMN40752635	PP747692	hCoV-19/USA/WI-UW-15486/2024
ATFGKWPAZL	SAMN40752634	PP747796	hCoV-19/USA/WI-UW-15485/2023
NO-BARCODE-TIZZY	SAMN40752633	PP747762	hCoV-19/USA/WI-UW-15484/2023
No-BARCODE-7	SAMN40752632	PP747803	hCoV-19/USA/WI-UW-15483/2023
No-Barcode-5-ihealth	SAMN40752631	PP747707	hCoV-19/USA/WI-UW-15482/2023
No-Barcode-5-BN	SAMN40752630	PP747800	hCoV-19/USA/WI-UW-15481/2023
ATPXOOQ032	SAMN40752629	PP747728	hCoV-19/USA/WI-UW-15480/2023
ATZ8EZY7IR	SAMN40752628	PP747782	hCoV-19/USA/WI-UW-15479/2023
ATZ0NRXDCK	SAMN40752627	PP747789	hCoV-19/USA/WI-UW-15478/2023
ATWJ2D0980	SAMN40752626	PP747742	hCoV-19/USA/WI-UW-15477/2023
ATUKMG6K7J	SAMN40752625	PP747785	hCoV-19/USA/WI-UW-15476/2023
ATPWR5IE4D	SAMN40752624	PP747695	hCoV-19/USA/WI-UW-15475/2023
ATPVOTRGYM	SAMN40752623	PP747802	hCoV-19/USA/WI-UW-15474/2023
ATNTDGQ0ZV	SAMN40752622	PP747778	hCoV-19/USA/WI-UW-15473/2023
ATM53BCTFV	SAMN40752621	PP747737	hCoV-19/USA/WI-UW-15472/2023
ATJR9OSFWD	SAMN40752620	PP747792	hCoV-19/USA/WI-UW-15471/2023
ATJ0KWF0TS	SAMN40752619	PP747739	hCoV-19/USA/WI-UW-15470/2023
ATFX9LZCHR	SAMN40752618	PP747713	hCoV-19/USA/WI-UW-15469/2023
ATCXJM1DBO	SAMN40752617	PP747790	hCoV-19/USA/WI-UW-15468/2023
ATBZPSGVAB	SAMN40752616	PP747795	hCoV-19/USA/WI-UW-15467/2023
AT96YQ9U13	SAMN40752615	PP747791	hCoV-19/USA/WI-UW-15466/2023
AT4VBM7WQH	SAMN40752614	PP747772	hCoV-19/USA/WI-UW-15465/2023
AT4J8ARPEG	SAMN40752613	PP747721	hCoV-19/USA/WI-UW-15464/2023
AT3ZC8GJPW	SAMN40752612	PP747767	hCoV-19/USA/WI-UW-15463/2023
AT0RH4DFUV	SAMN40752611	PP747779	hCoV-19/USA/WI-UW-15462/2023
No-barcode-4	SAMN40752610	PP747735	hCoV-19/USA/WI-UW-15461/2023
No-barcode-2	SAMN40752609	PP747717	hCoV-19/USA/WI-UW-15460/2023
ATZYNED0C2	SAMN40752608	PP747766	hCoV-19/USA/WI-UW-15459/2023
ATXXSAVO9F	SAMN40752607	PP747741	hCoV-19/USA/WI-UW-15458/2023
ATV6AW7859	SAMN40752606	PP747727	hCoV-19/USA/WI-UW-15457/2023
ATUZ85D2VU	SAMN40752605	PP747722	hCoV-19/USA/WI-UW-15456/2023
ATTYI7D5JH	SAMN40752604	PP747745	hCoV-19/USA/WI-UW-15455/2023
ATTYEJSDWY	SAMN40752603	PP747703	hCoV-19/USA/WI-UW-15454/2023
ATTVOS73K4	SAMN40752602	PP747694	hCoV-19/USA/WI-UW-15453/2023
ATSMWG98PK	SAMN40752601	PP747773	hCoV-19/USA/WI-UW-15452/2023
ATRX649RNA	SAMN40752600	PP747783	hCoV-19/USA/WI-UW-15451/2023
ATQN1DOYOX	SAMN40752599	PP747746	hCoV-19/USA/WI-UW-15450/2023
ATPDK4X5OH	SAMN40752598	PP747801	hCoV-19/USA/WI-UW-15449/2023
ATP8WMDMZK	SAMN40752597	PP747788	hCoV-19/USA/WI-UW-15448/2023
ATNTXE6UZQ	SAMN40752596	PP747756	hCoV-19/USA/WI-UW-15447/2023
ATNB4UEUAK	SAMN40752595	PP747709	hCoV-19/USA/WI-UW-15446/2023
ATMYVE1QEK	SAMN40752594	PP747758	hCoV-19/USA/WI-UW-15445/2023
ATKR3E8K57	SAMN40752593	PP747768	hCoV-19/USA/WI-UW-15444/2023
ATJUCHFV8Z	SAMN40752592	PP747799	hCoV-19/USA/WI-UW-15443/2023
ATG75FDZ90	SAMN40752591	PP747780	hCoV-19/USA/WI-UW-15442/2023
ATG0HMTYYE	SAMN40752590	PP747754	hCoV-19/USA/WI-UW-15441/2023
ATEHBAOC25	SAMN40752589	PP747747	hCoV-19/USA/WI-UW-15440/2023
ATCZTKHDF4	SAMN40752588	PP747718	hCoV-19/USA/WI-UW-15439/2023
ATBW04JJJ9	SAMN40752587	PP747753	hCoV-19/USA/WI-UW-15438/2023
ATBH4IK27M	SAMN40752586	PP747716	hCoV-19/USA/WI-UW-15437/2023
ATALAP7D6L	SAMN40752585	PP747704	hCoV-19/USA/WI-UW-15436/2023
ATAJAL9JG4	SAMN40752584	PP747712	hCoV-19/USA/WI-UW-15435/2023
ATACET5NJ8	SAMN40752583	PP747763	hCoV-19/USA/WI-UW-15434/2023
ATA5WLTAK0	SAMN40752582	PP747757	hCoV-19/USA/WI-UW-15433/2023
AT9E5WUVE3	SAMN40752581	PP747751	hCoV-19/USA/WI-UW-15432/2023
AT8DP3WGHB	SAMN40752580	PP747706	hCoV-19/USA/WI-UW-15431/2023
AT5M1NUPQS	SAMN40752579	PP747719	hCoV-19/USA/WI-UW-15430/2023
AT1REAXVVZ	SAMN40752578	PP747804	hCoV-19/USA/WI-UW-15429/2023
AT9M50GXL5	SAMN40752577	PP747784	hCoV-19/USA/WI-UW-15428/2023
AT7V74R0RW	SAMN40752576	PP747715	hCoV-19/USA/WI-UW-15427/2023
ATHXJM6OTS	SAMN40752575	PP747794	hCoV-19/USA/WI-UW-15426/2023

Internal ID	SRA accession no.	GenBank accession no.	GISAID virus name
AT7ZH839N8	SAMN40752574	PP747786	hCoV-19/USA/WI-UW-15425/2023
AT8X5RUNJL	SAMN40752573	PP747710	hCoV-19/USA/WI-UW-15424/2023
ATRBJD97CM	SAMN40752572	PP747736	hCoV-19/USA/WI-UW-15423/2023
ATF5O133LQ	SAMN40752571	PP747726	hCoV-19/USA/WI-UW-15422/2023
ATUQ7230RN	SAMN40752570	PP747731	hCoV-19/USA/WI-UW-15421/2023
AT0TFNDWZG	SAMN40752569	PP747724	hCoV-19/USA/WI-UW-15420/2023
ATCLJ5EIQG	SAMN40752568	PP747725	hCoV-19/USA/WI-UW-15419/2023
AT5UTIINHL	SAMN40752567		hCoV-19/USA/WI-UW-15418/2023
AT06HSAZ8J	SAMN40752566	PP747771	hCoV-19/USA/WI-UW-15417/2023
ATBSEDOKCN	SAMN40752565	PP747761	hCoV-19/USA/WI-UW-15416/2023
ATGRM0X3NG	SAMN40752564	PP747797	hCoV-19/USA/WI-UW-15415/2023
ATQC4UXFHL	SAMN40752563	PP747720	hCoV-19/USA/WI-UW-15414/2023
ATU2UEZGUU	SAMN40752562	PP747755	hCoV-19/USA/WI-UW-15413/2023
ATKCPI0YMU	SAMN40752561	PP747787	hCoV-19/USA/WI-UW-15412/2023
ATRUPA5DJ5	SAMN40752560	PP747732	hCoV-19/USA/WI-UW-15411/2023
ATFFPBDGC7	SAMN40752559	PP747777	hCoV-19/USA/WI-UW-15410/2023
AT25TA545F	SAMN40752558	PP747729	hCoV-19/USA/WI-UW-15409/2023
ATX2RLNE3M	SAMN40752557	PP747699	hCoV-19/USA/WI-UW-15408/2023
ATAC3S9AX4	SAMN40752556	PP747759	hCoV-19/USA/WI-UW-15407/2023
Lib-RAT-5-CC	SAMN40752555	PP747752	hCoV-19/USA/WI-UW-15406/2023
Lib-RAT-4-CC	SAMN40752554	PP747748	hCoV-19/USA/WI-UW-15405/2023
Lib-RAT-2-CC	SAMN40752553	PP747691	hCoV-19/USA/WI-UW-15404/2023
Lib-RAT-10-CC	SAMN40752552	PP747714	hCoV-19/USA/WI-UW-15403/2023

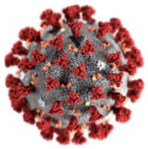

WISCONSIN
UNIVERSITY OF WISCONSIN–MADISON

Be part of a COVID-19 study!

We are collecting SARS-CoV-2 (COVID-19) positive at-home rapid antigen tests to identify the virus within them. Researchers across the world are doing this identification process so we can monitor the variants circulating and learn more about the evolution of COVID-19. This will help us be able to predict or prevent its spread in the future.

How you can help

Participating in this effort is simple!

1. Put your positive rapid antigen test into the pre-paid envelope.
2. Using your phone's built-in camera, please scan the QR code in the Ziploc, which will take you to a website that records the date and location when you scan the code. Your phone's camera should already have the ability to recognize QR codes. You should not need to download an app for this.
3. If you have trouble scanning the QR code, you can visit http://rat.wisc.edu and type your code in manually.
4. Please place the barcode back inside the Ziploc before sealing the envelope.
5. Drop the sealed envelope into a mailbox. Thank you!

We will protect your privacy

While we will not collect any personal information about you, scanning the QR code will record your location and the time you scanned the code, which is considered to be private information. These data will help us to determine when and where the virus was present in the community. These data will be stored in a secure database with access only for the scientists on the study. After we sequence your sample and determine the variant, we will upload the sequence information, date of scan, and in which county the QR code was scanned to national databases. These national databases (NCBI Virus and NCBI SRA) are a resource for all scientists doing COVID research and contain millions of sequences.

More about this project

Throughout the COVID pandemic, the David O'Connor group at the University of Wisconsin has been sequencing COVID from nasal swabs to determine variants active in our community. As rapid antigen tests have become the main type of COVID test, we have developed methods to sequence the virus directly from these tests. Our objective is to continue sequencing samples in order to understand the variants present in the community. If you have questions about this project, please contact us at 608-890-0847. You can look up some of our work at: https://dho.pathology.wisc.edu/.

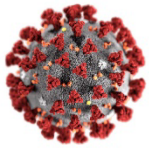

WISCONSIN
UNIVERSITY OF WISCONSIN–MADISON

¡Forma parte de nuestro estudio acerca del COVID-19!

Estamos recolectando pruebas rápidas de antígeno positivas para SARS-CoV-2 con el propósito de identificar qué tipo de virus contienen. Diferentes investigadores alrededor del mundo realizan esta práctica de identificación para monitorear las diferentes variantes del virus que circulan en la comunidad y para conocer más acerca de la evolución de COVID-19. Esto nos ayudará a predecir y prevenir su propagación.

¿Cómo puedes ayudar?

¡Participar en este esfuerzo es sencillo!

1. Coloca tu prueba rápida de antígeno positiva para SARS-CoV-2 en el sobre pre-paga-do.
2. Utilizando la cámara integrada de tu teléfono, escanea el código QR en la bolsa ziploc, lo cual te llevará a un sitio web que registra la fecha y la ubicación cuando escaneas el código. La cámara de tu teléfono ya debería tener la capacidad de recon-ocer códigos QR. No deberías necesitar descargar una aplicación para esto.
3. Ponga el código de barras dentro de la bolsa Ziploc antes de cerrar el sobre.
4. Deposita el sobre sellado en un buzón.

Protegeremos su privacidad

A pesar de que no recopilaremos su información personal, el escanear el código registrará su ubicación y fecha en la que escaneó el código, lo cual es considerado información privada. Estos datos nos ayudarán a determinar cuándo y dónde el virus estuvo presente en la comunidad. Esta información será almacenada en una base de datos segura cuyo acceso será restringido únicamente a los científicos a cargo del estudio. Luego de secuenciar su muestra y determinar la variante, enviaremos a las bases de datos nacionales la información de la secuencia, fecha del escaneo del código y el país de donde el código fue escaneado. Estas bases de datos nacionales (NCBI Virus y NCBI SRA) son un recurso para muchos científicos que investigan el COVID y contienen millones de secuencias.

Más acerca de este proyecto

A lo largo de la pandemia del COVID, el grupo de investigación de David O'Connor en la Universidad de Wisconsin ha estado secuenciando COVID proveniente de hisopos nasales para determinar las variantes activas en nuestras comunidades. Dado a que las pruebas de antígenos se han convertido en el tipo de prueba principal para detectar COVID, hemos desarrollado métodos para secuenciar el virus directamente de estas pruebas. Nuestro objetivo es continuar secuenciando muestras para así poder entender las variantes que predominan en la comunidad. De tener preguntas acerca de este proyecto, por favor contáctenos al 608-890-0847. Puede encontrar parte de nuestro trabajo en: https://dho.pathology.wisc.edu/.

Appendix Figure. Contents of the flyer attached to each rapid antigen test collection packet. This flyer explains the goal of the study and provides instructions for those who want to participate in English (first page) and Spanish (second page).
